# Pituitary Xanthogranuloma Causing Sellar Mass in 2 Children With Hypopituitarism and Elevated Inflammatory Markers

**DOI:** 10.1210/jcemcr/luaf260

**Published:** 2025-10-31

**Authors:** Madalin Berra, Laurel Gordon, Nikta Forghani

**Affiliations:** University of California Irvine, Orange, CA 92868, USA; Children’s Hospital of Los Angeles, Los Angeles, CA 90027, USA; Children’s Hospital of Orange County, Orange, CA 92868, USA

**Keywords:** xanthogranuloma, cholesterol granuloma, sellar mass, hypopituitarism

## Abstract

Pituitary xanthogranulomas represent a rare group of inflammatory pituitary masses. The prevalence of these lesions is not clearly defined. These lesions, also known as cholesterol granulomas, are diagnosed on histopathological findings including cholesterol clefts, hemosiderin deposits, foamy macrophages, and necrosis. They often present with pituitary hormone deficiencies and have a low recurrence rate following gross total resection. We describe 2 patients presenting with headaches, polyuria, polydipsia, and elevated erythrocyte sedimentation rate. One patient had a history of Rathke cleft cyst (RCC). Magnetic resonance imaging identified sellar masses, and they were subsequently diagnosed with varying degrees of pituitary hormone deficiencies preoperatively. They underwent transsphenoidal mass resection with pathology showing xanthogranuloma. Postoperatively they have panhypopituitarism including arginine vasopressin deficiency and no recurrence of the lesion. Inflammatory causes of pituitary lesions should remain on the differential, especially in patients with a history of RCC. More research is needed to identify the prevalence of xanthogranulomatous pituitary lesions and better understand the pathophysiology of their formation. The finding of elevated inflammatory markers, not usually seen in other pituitary masses or previously documented in the literature in cases of xanthogranulomatous pituitary lesions, could reflect a clinical clue when evaluating patients with pituitary lesions.

## Introduction

Xanthogranuloma, also known as a cholesterol granuloma, is a rare pituitary lesion, minimally described in the pediatric literature. The precipitating etiology for the consequent inflammatory reaction remains unknown in many cases, although it is generally thought to arise as a response to keratin or mucin potentially released by damage to an underlying epithelial lesion such as craniopharyngioma, adenoma, or Rathke cleft cyst (RCC) [1, 2].

Due to a lack of specific characteristics on radiologic imaging, similarity in presentation to a variety of other pituitary tumors, and primary diagnosis by histology, most xanthogranulomas are undiagnosed until resection is completed. Cases of xanthogranuloma have been reported both in the pediatric and adult populations; however, overall cases are rare and estimated to compose only about 0.6% to 2% of all pituitary tumors [3, 4]. Among pediatric cases, there has been variability in clinical presentation, although most common symptoms included headache, followed by polyuria and polydipsia and growth retardation [5].

## Case Presentation

### Patient 1

A 10-year-old previously healthy girl presented to the emergency department with lethargy and worsening headaches. She had a 6-month history of frequent headaches, marked polydipsia, polyuria, nocturia, decreased appetite, slowed weight gain, and linear growth (2.8 cm/year). She was prepubertal. Her initial exam revealed no neurologic deficits, and ophthalmologic exam showed no papilledema or visual deficits.

### Patient 2

**Figure 1.**
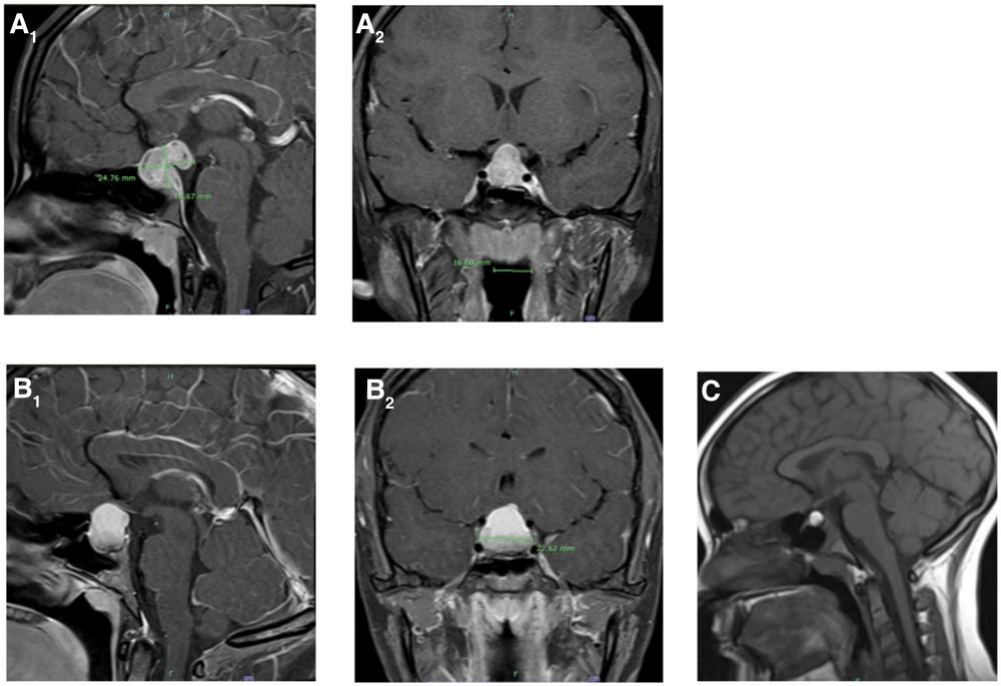
Preoperative imaging studies. (A**)** Patient 1 MRI sagittal and coronal views at diagnosis showing a 1.7 × 2.5 × 1.9 cm heterogeneously enhancing mass involving the sella and the suprasellar regions. It is isointense on the T1-weighted images and hypointense on the T2-weighted images with remodeling of the dorsum sella and absent posterior pituitary bright spot**. (**B) Patient 2 MRI sagittal and coronal views at diagnosis showing a 1.6 × 2.2 × 2.3 cm heterogeneously T1 hyperintense lesion expanding the sella with suprasellar expansion, spuriously deviated infundibulum, and splaying of the prechiasmatic optic nerves**. (**C) Patient 2 noncontrast MRI 5 years prior to diagnosis showing 1.4 cm Rathke cleft cyst. Abbreviation: MRI, magnetic resonance imaging.

A 15-year-old female with chronic headaches presented with acute worsening of headaches and aphasia. Due to her chronic headache history, at age 10 magnetic resonance imaging (MRI) was completed that demonstrated a 1.4 cm RCC (see Fig. 1). No follow-up MRI had ever been done. She also noted a years’ long history of polyuria and polydipsia but had acclimated to this lifestyle. She had attained a normal adult height for the family and appeared to have some pubertal progression but had never reached menarche. Ophthalmologic evaluation was normal with no papilledema or visual deficits, and her aphasia resolved soon after admission.

## Diagnostic Assessment

### Patient 1

In the months leading up to her presentation, patient 1 had multiple laboratory evaluations pursuing her presenting symptoms without a cause identified, but no hormonal studies were ever done. Her only notable findings were an elevated erythrocyte sedimentation rate (ESR) on 2 separate occasions [41 mm/hr and then 50 mm/hr 6 weeks later (reference range: 0-20)]. Due to the persistent headaches, MRI was completed and revealed a 1.7 × 2.5 × 1.9 cm mass involving the sella and suprasellar region, obliterating the infundibulum, and also notable for absence of a posterior pituitary bright spot (see Fig. 1). Pituitary hormone evaluation was completed and significant for mildly elevated prolactin and low free T4 and IGF-1. Her random cortisol was also low, and response to a low/high dose cosyntropin stimulation test was interpreted as inadequate. Her initial evaluation for arginine vasopression (AVP) deficiency was unremarkable (and her symptoms of polyuria and polydipsia had in fact dissipated in the few months leading up to diagnosis), but there was a high suspicion that AVP deficiency would become unmasked when pituitary replacement was started. Finally, her ESR was now elevated to 71 mm/hr. See Table 1 for a summary of laboratory results. Oncologic evaluation was notable for normal serum and cerebrospinal fluid alpha-fetoprotein and human chorionic gonadotropin. A skeletal survey was negative for any lytic lesions.

**Table 1.** Preoperative laboratory findings

	Patient 1 result (reference range)	Patient 2 result (reference range)
Free T4	0.66 ng/dL (0.89-1.37 ng/dL) [SI: 8.5 pmol/L (11.5-17.6 pmol/L)]	0.7 ng/dL (0.89-1.37 ng/dL) [SI: 9 pmol/L (11.5-17.6 pmol/L)]
TSH	1.38 uIU/mL (0.7-4.17 uIU/mL)	1.5 uIU/mL (0.7-4.17 uIU/mL)
IGF-1	72 ng/mL (85-306 ng/mL) [SI: 9.41 nmol/L (11.11-40 nmol/L)]	63 ng/mL (194-516 ng/mL) [SI: 8.23 nmol/L (25.36-67.45 nmol/L)]
ACTH	11 pg/mL (6-48 pg/mL) [SI: 2.42 pmol/L (1.3-10.57 pmol/L)]	11 pg/mL (6-48 pg/mL) [SI: 2.42 pmol/L (1.3-10.57 pmol/L)]
Prolactin	27.2 µg/L (4-23 µg/L)	29.8 µg/L (4-23 µg/L)
ESR (mm/hr)	71 mm/h (0-20 mm/h)	21 mm/h (0-20 mm/h)
CRP	<5 mg/L (0-10 mg/L) [<47.6 nmol/L (0-95.2 nmol/L)]	<5 mg/L (0-10 mg/L) [<47.6 nmol/L (0-95.2 nmol/L)]
Cosyntropin stimulation test		
Baseline cortisol	1.4 µg/dL (5-27 µg/L) [SI: 38.6 nmol/L (137.9-744.8 nmol/L)]	5.8 µg/dL (5-27 µg/L) [SI: 160.2 nmol/L (137.9-744.8 nmol/L)]
1 hour post-1 mcg cosyntropin n	1 µg/dL [SI: 17.5 nmol/L]	9.9 µg/dL [SI: 273.1 nmol/L]
1 hour post-250 mcg cosyntropin	18.3 µg/dL [SI: 496.5 nmol/L]	19 µg/dL [SI: 524.1 nmol/L]

Abbreviations: CRP, C-reactive protein; ESR, erythrocyte sedimentation rate.

### Patient 2

Due to patient 2’s worsening headaches, MRI was done, which showed a heterogeneous T1 hyperintense lesion expanding the sella with suprasellar expansion measuring 1.6 × 2.2 × 2.3 cm. The infundibulum also appeared deviated superiorly, and there was splaying of the prechiasmatic optic nerves (see Fig. 1). Pituitary hormones were completed and significant for elevated prolactin and low free T4 and IGF-1 levels. Her morning cortisol was adequate, but she also underwent a low/high cosyntropin stimulation test that was interpreted as partially adequate, and therefore no replacement was started at that time. See Table 1 for a summary of laboratory values. She was noted to have polyuria and polydipsia, but after a 10-hour water deprivation, her sodium was 137 mMol/L, serum osmolality was 292 mOsm/kg H2O, and urine osmolality was dilute for a fasting state at 209 mOsm/kg H2O. Ultimately, she was diagnosed with central hypothyroidism, partial adrenal insufficiency, and partial AVP deficiency, but given clinical stability and asymptomatic state, replacement was held given impending surgical plan. Her ESR was elevated at 21 mm/hr. Oncologic evaluation was unremarkable, including normal serum alpha-fetoprotein and human chorionic gonadotropin.

## Treatment

### Patient 1

Preoperatively patient 1 started physiologic cortisol replacement with hydrocortisone and subsequently initiated levothyroxine. She had near resolution of low energy, nausea, and anorexia that she had experienced in the year prior to presentation within 1 week of replacement, reflecting clinical response. After 72 hours on hydrocortisone replacement, her urine output rose to 5.7 cc/kg/hr (urine output over 24 hours documented at 3840 mL), reflecting an unmasking of underlying AVP deficiency, so oral desmopressin was started with good response. She underwent transsphenoidal sellar/suprasellar mass resection in conjunction with otolaryngology and neurosurgery. During the procedure, after the dura was opened, there was drainage of yellow fluid. This was sent for intraoperative pathology evaluation and was noted to contain inflammatory cells. The mass was removed in its entirety.

### Patient 2

Patient 2 started hydrocortisone stress dosing preoperatively. She underwent transsphenoidal sellar/suprasellar mass resection in conjunction with otolaryngology and neurosurgery. A tan-colored mass was appreciated and had drainage of chronic appearing liquid and semisolid blood products, suggesting prior internal hemorrhage. The mass was then removed in its entirety.

## Outcome and Follow-up

### Patient 1

Preliminary pathology was negative for malignancy and significant for a necroinflammatory process. Sections reveal fibrotic tissue showing extensive foci of necrotizing basophilic debris with associated histiocytic and focally giant cell reaction, accompanied by neutrophils located within the debris as well as mature lymphoid cells and only occasional plasma cells. BRAF and SALL4 staining were negative. No organisms were detected on special stains and by PCR analysis. Her case was discussed among endocrinology, neurosurgery, oncology, and pathology and ultimately was felt to be most consistent with a pituitary xanthogranuloma.

**Figure 2.**
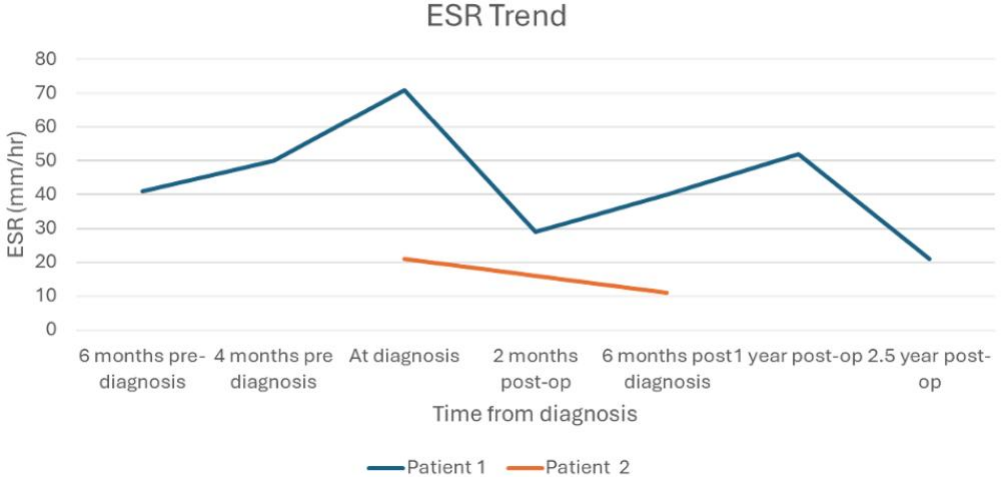
Erythrocyte sedimentation rate trend.

Her postoperative course was notable for overt AVP deficiency requiring a continuous vasopressin infusion but was otherwise uncomplicated. Two years later, the patient continues to have persistent hypopituitarism on desmopressin, hydrocortisone, and levothyroxine replacement. She had no pubertal development with undetectable gonadotropin levels, suggesting hypogonadotropic hypogonadism, and growth remained very poor. She started GH and estrogen replacement with subsequent improvement in growth velocity. Serial MRIs show stable postoperative changes and no mass regrowth. Her ESR level, although initially fluctuated, has now normalized (see Fig. 2).

### Patient 2

Patient 2’s surgical pathology report revealed a RCC with xanthogralulomatous changes and hematoma with focal squamous metaplasia and calcification. Postoperatively, she had increased urine output at 6 mL/kg/hr over 24 hours, reflecting overt AVP deficiency, which quickly resolved and did not require intervention. Her preoperative adrenal evaluation (Table 1) warranted hydrocortisone replacement perioperatively, but she eventually weaned off. Repeat testing revealed global hypopituitarism with notably low morning cortisol and low free T4 and IGF-1 levels (Table 2), requiring replacement with hydrocortisone, levothyroxine, and GH. She remained amenorrheic postoperatively with low gonadotropins, so estrogen replacement was also initiated. Finally, she continues to have partial AVP deficiency with polyuria, polydipsia, and inability to adequately concentrate urine after overnight fasting, but she has never developed hypernatremia and remains off desmopressin. Follow-up MRI shows stable postoperative changes. Her ESR has normalized (see Fig. 2).

**Table 2.** Postoperative laboratory findings

	Patient 1 result (reference range)	Patient 2 result (reference range)
Free T4	0.8 ng/dL (0.89-1.37 ng/dL)*^a^* [SI: (pmol/L (11.5-17.6 pmol/L)]	0.42 (0.89-1.37 ng/dL) [SI: (pmol/L (11.5-17.6 pmol/L)]
IGF-1	62 ng/mL (85-306 ng/mL) [SI: 8.1 nmol/L (11.11-40 nmol/L)]	39 ng/mL (194-516 ng/mL) [SI: 5.1 nmol/L (25.36-67.45 nmol/L)]
Am cortisol	*^a^*	0.2 µg/dL (6.2-19.4 µg/dL) [SI: 5.51 nmol/L (171-535.17 nmol/L)
Prolactin	<2 µg/L (4-23 µg/L)	2.2 µg/L (4-23 µg/L)
ESR	19 mm/h (0-20 mm/h)	11 mm/h (0-20 mm/h)

Abbreviation: ESR, erythrocyte sedimentation rate.

^*a*^Patient was already on cortisol and thyroid hormone replacement.

## Discussion

Inflammatory lesions are an often overlooked but important cause of sellar mass presenting with pituitary hormone deficiency in pediatric patients. These lesions can be caused by a primary inflammatory process or as a secondary reaction to a preexisting pituitary mass [1]. The most common type from either origin is hypophysitis, of which the origin can be lymphocytic (68%), granulomatous (20%), xanthomatous (3%), or rarely mixed type (such as xanthogranulomatous) [6]. Less common are xanthogranulomas, also known as cholesterol granulomas. They can occur in multiple intracranial and extracranial locations, with the sellar region being 1 of the least common [1].

Inflammatory lesions present similarly to other sellar masses, with headache, vision changes, and pituitary hormone deficiencies. Contrastingly, as was seen in our cases, these lesions have a much higher predominance for AVP deficiency, which is a rare presenting symptom in adenomas and craniopharyngiomas [6-8].

These lesions require tissue diagnosis. Histopathological findings include cholesterol clefts, hemosiderin deposits, giant cells, foamy macrophages, and necrosis [1, 4]. The pathogenesis has been debated but is thought to be an inflammatory reaction following rupture, leakage, hemorrhage, or apoplexy of a preexisting pituitary mass [1, 2, 4]. However, for many patients, including our first patient, if there is no earlier imaging available for comparison or findings on pathology report to suggest underlying pathology, it may not be possible to definitively determine an origin. In our second case, the known history of RCC was informative.

There is little reported about the role of monitoring inflammatory markers in these patients. Our first patient had a significantly elevated ESR prior to diagnosis, which improved postoperatively but took 2 years to normalize despite her surgery and lack of evidence of recurrence. Our second patient also had a mildly elevated ESR prior to surgery that normalized soon after. Both patients had normal C-reactive protein levels. Due to the lack of available data, we are unable to determine how these markers correlate to disease status given their stable imaging studies. Further research on this topic would be beneficial in understanding the correlation of inflammatory markers with risk of recurrence of xanthogranulomas or correlation with ongoing hormonal deficiencies following tumor resection. Perhaps at a minimum, finding an elevated ESR during the evaluation of a new pituitary mass could help guide diagnosis.

Our cases exemplify the importance of keeping inflammatory lesions on the differential for pituitary lesions. More research is needed to identify the prevalence of xanthogranulomatous pituitary lesions and to better understand the pathophysiology of their formation and their potential for resolution.

## Learning Points

- In a patient with a history of RCC, xanthogranuloma should be a consideration if there is new evidence of neurologic concerns or hormonal deficiency.
- Inflammatory lesions remain an important differential diagnosis when evaluating a pediatric patient with sellar mass.
- Elevated inflammatory markers may be useful in the workup of pituitary masses.

## Contributors

M.B, L.G., and N.F. were involved in the diagnosis and management of this patient and manuscript submission.

## Data Availability

Data sharing is not applicable to this article as no datasets were generated or analyzed during the current study.
